# Near-complete genome sequence of the opportunistic yeast pathogen *Nakaseomyces glabratus* environmental isolate D77_3

**DOI:** 10.1128/mra.00367-25

**Published:** 2025-06-25

**Authors:** Roumen Dimitrov, Nicolò Tellini, Matteo De Chiara, Gianni Liti, Dilnora Gouliamova

**Affiliations:** 1 Institute of Microbiology, Bulgarian Academy of Sciences, Sofia, Bulgaria; 2 Institute of Research on Cancer and Ageing of Nice (IRCAN), Faculté de Médecine, Nice, France; University of California Riverside, Riverside, California, USA

**Keywords:** insect associated yeasts, pathogens, *Nakaseomyces*

## Abstract

We present the near-complete genome sequence of *the Nakaseomyces glabratus* environmental isolate D77_3, obtained from the gut of the beetle *Oxythyrea funesta* (Coleoptera: Cetoniinae), collected in Sofia, Bulgaria. This genome was *de novo* assembled and adds valuable data on *N. glabratus* diversity.

## ANNOUNCEMENT

*Nakaseomyces glabratus* (formerly *Candida glabrata*) is an opportunistic yeast pathogen causing fatal bloodstream infections (candidemia) in immunocompromised adults (1). We report the near-complete genome of environmental isolate D77_3, obtained from the dissected gut of the beetle *Oxythyrea funesta* collected in Sofia, Bulgaria. This genome contributes to understanding the evolutionary mechanisms enabling *N. glabratus* to adapt to diverse environments, including its shift from environmental to human-associated niches. Methods for the strain isolation and identification and accession numbers of ribosomal markers were described previously (2). The isolate is deposited in the Bulgarian National Collection of Microorganisms and Cell Cultures, under accession number NIMCC 9134. The strain is preserved in lyophilized form and maintained at +4°C. It is available from the authors upon request.

The strain was cultured on YPGA medium prior to DNA extraction. Genomic DNA was extracted using the Maxwell RSC Tissue DNA Kit (Promega). For PacBio sequencing, 8  µg DNA was sheared (Covaris) and size-selected with AMPurePB beads. Libraries were prepared using the SMRTbell Template Prep Kit 1.0 (PacBio), and subreads fully contained within other subreads or showing abnormal overlaps were removed. A total of 145,092 PacBio long reads were generated. For Illumina sequencing, 2  µg DNA was sheared (Covaris), end-repaired, A-tailed, and ligated with TruSeq DNA UD adapters (Illumina). Libraries were quantified by qPCR using the KAPA Library Quantification Kit (Roche), and quality was assessed using a Bioanalyzer (Agilent). After adapter trimming and quality filtering (Phred score ≥Q20), 16,454,074 Illumina reads were retained, with 95.88% of bases ≥ Q30.

*De novo* assembly of PacBio reads was performed using HGAP3 (v3.0) (3), followed by three polishing rounds with Pilon v1.21 (4) using Illumina data. Post-polishing, 16,440,965 Illumina reads mapped to the assembly, covering 99.96% of the genome at an average depth of 182.27×. Completeness was assessed with BUSCO v3.0, using eukaryota_odb9 and *Saccharomyces cerevisiae* data sets (5) . Assembly quality was evaluated using QUAST v5.0.2 (6) by comparison to the *N. glabratus* reference genome (GCF_000002545.3_ASM254v2), confirming 99.13% completeness.

Telomeric repeats were detected using the motif TCTGGGTGCTGTGGGG and its reverse complement. Contigs 1, 5, and 8 contained repeats at both ends, indicating complete chromosomes. Alignment confirmed other contigs mostly correspond to full chromosomes, except for terminal telomeric repeats. Structural rearrangements between chromosomes 4 and 12, involving contigs 2, 12, and 13, were identified.

The final assembly totaled 12.65 Mb, with a median PacBio coverage of 76×, an average GC content of 38.98%, and 15 contigs (6 larger than 1,000 bp; N50 = 1,058,520 bp and 9 smaller contigs < 1,000 bp).

## Data Availability

The genome of *N. glabratus* isolate D77_3 has been deposited in DDBJ/ENA/GenBank under BioProject PRJNA802031 with accession number JAKMBM000000000.1. The version described in this paper is version JAKMBM000000000.1. Raw sequencing data (Illumina and PacBio) have been deposited in NCBI SRA under accession numbers SRR33245981 and SRR33245982.

## AFTER EPUB

*[This article was published on 25 June 2025 with an error in the author byline. The byline was corrected in the current version, posted on 1 July 2025.]*
